# What Should General Practice Trainees Learn about Atopic Eczema?

**DOI:** 10.3390/jcm4020360

**Published:** 2015-02-12

**Authors:** Deepani Munidasa, Antonia Lloyd-Lavery, Susan Burge, Tess McPherson

**Affiliations:** 1 Department of Dermatology, Polonnaruwa General Hospital, Polonnaruwa, 51000, Sri Lanka; E-Mail: deepanimunidasa@yahoo.com; 2 Department of Dermatology, Oxford University Hospitals NHS Trust, Oxford, OX3 7LJ, UK; E-Mail: tess.mcpherson@ouh.nhs.uk; 3 Nuffield Department of Medicine, University of Oxford, Oxford, OX3 9DS, UK; E-Mail: sue.burge@ndm.ox.ac.uk

**Keywords:** atopic eczema, dermatology curriculum, RCGP curriculum, patient involvement medical education, GP training

## Abstract

Effective atopic eczema (AE) control not only improves quality of life but may also prevent the atopic march. The Royal College of General Practitioners’ (RCGP) curriculum does not currently provide specific learning outcomes on AE management. We aimed to gain consensus on learning outcomes to inform curriculum development. A modified Delphi method was used with questionnaires distributed to gather the views of a range of health care professionals (HCPs) including general practitioners (GPs), dermatologists, dermatology nurses and parents of children with AE attending a dedicated paediatric dermatology clinic. Ninety-one questionnaires were distributed to 61 HCPs and 30 parents; 81 were returned. All agreed that learning should focus on the common clinical features, complications and management of AE and the need to appreciate its psychosocial impact. Areas of divergence included knowledge of alternative therapies. Parents felt GPs should better understand how to identify, manage and refer severe AD and recognized the value of the specialist eczema nurse. Dermatologists and parents highlighted inconsistencies in advice regarding topical steroids. This study identifies important areas for inclusion as learning outcomes on AE management in the RCGP curriculum and highlights the importance of patients and parents as a valuable resource in the development of medical education.

## 1. Introduction

Atopic eczema is a chronic, itchy skin condition that affects up to 20% of children in the UK. The disease has a considerable psychosocial impact on both patients and their families [1,2,3]. Schofield analysed contemporary surveillance data from the Weekly Returns Service of the Royal College of General Practitioners (GPs) and showed that skin conditions were the most frequent reason (24% of first time visits) for consultations in general practice and that 20% of children under 12 months old were diagnosed with atopic eczema [4].

The 2007 guidelines published by the National Institute for Health and Clinical Excellence (NICE) [5] state that “it is known that a lack of education about therapy leads to poor adherence, and consequently to treatment failure”. Furthermore, a lack of good information and support causes significant stress for patients and their families [6,7]. Education of health care professionals and patients improves management of atopic eczema [8] as this is a chronic illness with a significant impact on the quality of life of affected children and their families, often necessitating multiple and at times complex treatments. There is increasing evidence that atopic eczema control not only improves quality of life but prevents further atopic disease (the atopic march to asthma/allergic rhinitis) [9].

We aimed to identify what GP trainees should learn about atopic eczema. The RCGP curriculum does not provide any learning outcomes specifically related to atopic eczema [10]. The General Medical Council (GMC) has identified the particular importance of patient involvement in medical education and identifying ways to include patients at all levels of training is a current priority [11]. This research involves patients’ opinion in developing training curricula which is an area where there is very little published to date.

## 2. Methods and Participants

A literature search on Medline, PubMed and Google Scholar using the key term “atopic eczema” coupled separately with each of the key terms “GP Dermatology curriculum”, “expert patients”, “parent input” and “medical curriculum development” from the earliest available articles available through to September 2012 did not reveal any previous studies which had established the views of healthcare professionals or parents of children with atopic eczema about what GPs should learn about atopic eczema.

The Delphi method, which uses a panel of experts to achieve consensus on the answers to a number of questions, is a useful tool for developing curricula [12]. We used a modified Delphi study to explore the views of two panels on what a GP trainee should learn about atopic eczema: one composed of health care professionals and the other composed of parents of children with atopic eczema attending a dedicated paediatric dermatology clinic at the Children’s Hospital in Oxford.

We formulated one questionnaire for the healthcare professionals and another, written in non-technical language, for the parents. The items in the questionnaires were generated from the dermatology curriculum for GP trainees, published literature on the multifaceted impact of childhood eczema and a similar questionnaire used by one of the authors (Susan Burge) to define the medical undergraduate curriculum for psoriasis [13]. Each questionnaire contained a number of items under the broad categories of knowledge, management, practical skills and attitudes in relation to atopic eczema. The questionnaire for the parents contained additional questions on the psychosocial aspects of atopic eczema.

The questionnaires were first piloted with a panel of four dermatologists, a specialist dermatology nurse and five parents of children with atopic eczema, who were invited to comment on the content, clarity and wording. Their comments were incorporated into the final questionnaires.

The final questionnaire for the healthcare professionals containing 33 items was given to 22 dermatologists (8 teaching hospital consultants, 6 district general hospital consultants, 8 dermatology registrars), 6 GP trainees working in dermatology, 1 paediatric dermatology nurse, 2 paediatricians and 30 general practitioners including some GP trainers. These were either handed out in person or posted to them with a return envelope. All were asked to return the completed anonymous questionnaire by post. The final questionnaire for parents of children with chronic atopic eczema containing 30 items was handed out to 30 parents of children with atopic eczema attending the paediatric dermatology clinic and they were asked to return the completed anonymous questionnaire by post.

The panel were asked to accept, reject or question the inclusion of each item in the curriculum. Items for which a level of 75% consensus was reached would either be accepted or rejected. The panel were encouraged to supply additional comments.

## 3. Results

Ninety-one questionnaires (61 healthcare professionals and 30 parents) were distributed with 81 (89%) returns. Five of 30 parents and 5 of 30 GPs did not return the questionnaires. All returned questionnaires were completed correctly. It was possible to achieve sufficient consensus with a single round of questioning. To identify areas of divergence we also looked at the responses of health care professionals and parents separately.

Topics selected as appropriate for inclusion in the curriculum by both healthcare professionals and parents with a consensus level of 75% or more are included in Box 1.

Areas where opinions of parents and healthcare professionals diverged are listed in Box 2.

The panels agreed that learning should focus on the common clinical features, complications and management of atopic eczema. The panels also agreed that GP trainees should learn about the psychosocial impact of eczema.

Additional free text comments were also encouraged in the questionnaires. Health care professionals commented on the importance of knowing how to deal with anxieties about topical steroids and when to refer when these were not working. The free text comments of parents were particularly revealing and are listed in Box 3. These particularly highlighted the discrepancy in advice regarding the use and potency of topical steroids in general practice compared to the dermatology clinic.

Box 1 **Items accepted by 75% of healthcare professionals and parents of children with atopic eczema.**

**GP trainees should know:**

- The common physical signs of atopic eczema
- The different forms/presentations of atopic eczema
- What makes eczema worse
- The principles of treatment in general
- The principles of topical treatment thoroughly
- How to apply topical treatment, explain and prescribe appropriate amounts of creams and ointments
- The complications of atopic eczema (e.g., eczema herpeticum, impetigo)
- That different age groups and different areas of skin need different treatment
- The importance of explanation to patient/parent and reassurance
- When more advice on care is needed and when to refer to the specialist
- Where to ask for more information and help
- The importance of asking patients/parents about their hopes of treatment
- The importance of examining the skin in a sensitive and courteous manner
- The physical and psychological impact of atopic eczema on the patient
- The need to provide time for parent/patient to ask questions

Box 2 **Areas of divergence between healthcare professionals and parents of children with eczema.**

**GP trainees should know:**

- **The importance of the role of dermatology nurses**
  (Selected by 80% parents but only 64% of dermatologists and 32% of GPs)
- **The indications for more advanced (systemic) treatment**
  (Selected by 72% of parents but only 48% of healthcare professionals)
- **The importance of addressing associated problems such as allergies and asthma**
  (Selected by 84% parents but only 60.7% healthcare professionals)
- **About alternative and complementary therapies**
  (Selected by 68% of parents but only 0.1% of healthcare professionals)

Box 3 **Free text comments of parents of children with atopic eczema.**

- “GPs need to know when to refer and about when to use steroids of different strength.”
- “GPs tend to be dismissive about eczema. They should have a chart/list about all the treatments available and explain them to the parents. GPs tend to stick to hydrocortisone cream and would not consider stronger steroids even if needed.”
- “We saw a specialist who prescribed ointments to help relieve eczema. GPs say we shouldn’t be using ointments as much we are. I trust the specialist as they have seen far more serious cases of eczema. So GP should trust the info given to families is correct.”
- “There is a discrepancy between GP advice and hospital advice regarding steroid use. Repeat visits to GP can be frustrating when no treatment strategies are offered.”
- “I kept being told there is no cure for eczema and felt as if each time I was wasting my GP’s time. I had many appointments but ‘1% steroid cream’ was ineffective. Only when I saw a dermatologist and was prescribed Elocon that the eczema cleared up. I now only need to use it once a week and my little boy is very happy and so are we.”
- “The GP should have referred us to the specialist sooner. I'm not sure her knowledge of the treatment was accurate as we were only offered Fucidin H for months. An Emollient was prescribed but I was not taught how to use this.”
- “GPs in our experience, generally write a hurried prescription for yet another cream to send us on our way. The information sheet we received at the JR (hospital) offered more info than we have ever been given in our 6 years. I think that our GP should be able to discuss with the parents their problems, offer mutual support and refer to the specialist where necessary.”
- “My daughter had severe eczema. I was a given a cream and only repeatedly told that she will grow out of it. I was not taken seriously. My doctor needs to know how to identify bad disease and when to refer to the specialist.”
- “I was told to avoid dairy for no solid reason. This had big implications and did not help my child’s eczema. I think GPs should know more about food allergies in atopic eczema.”
- “Be open to solutions other than steroids, be willing to refer to specialists if required/requested.”

Seventy-two percent of parents but only 48% of healthcare professionals thought it important for GP trainees to know the indications for more advanced (systemic) treatment.

Eighty percent of parents (all of whom would have had support from a specialist nurse), 64% of dermatologists, but only 32% of GPs thought the atopic eczema curriculum should include learning about the role of dermatology nurses.

Sixty-eight percent of parents, in contrast to 0.1% of healthcare professionals, wanted GP trainees to know about alternative and complementary therapies in relation to atopic eczema.

Eighty-four percent of parents and 61% of healthcare professionals identified the importance of addressing associated atopic problems such as allergies and asthma.

## 4. Discussion

### 4.1. What Should GP Trainees Learn?

There was consensus between health care professionals and parents of children with atopic eczema that GP trainees should learn about the common clinical features, complications and management of atopic eczema.

The panels also agreed that the psychosocial impact of eczema should be appreciated, with parents particularly highlighting the need for trainees to be aware of the impact of eczema on the child’s social and physical activities. The physical discomfort and sleep disturbance as well as the stigma caused by eczema may interfere with the child’s emotional and social development, but is often underestimated.

In general the diagnosis of atopic eczema is not difficult, but many children with eczema are undertreated because of parents’ fears of corticosteroid safety, “steroid phobia”, or confusion about the potency of steroid prescribed [14,15,16]. The GP is the main source of information for most patients and their first point of contact for the management of atopic eczema [14], so it is crucial that trainees learn how to prescribe and advise on the use of topical corticosteroids and have sufficient knowledge to allay anxieties. The free hand comments section included in the questionnaires particularly highlighted the discrepancy in advice given regarding the use and potency of topical steroids in general practice compared to the dermatology clinic, identifying this as an important area for further training. Parents particularly identified the need for trainees to learn about the indications for systemic treatment, and both panels agreed that trainees should know when to refer to the dermatology clinic.

The divergence seen between healthcare professionals and parents on the importance of GP trainees knowing the indications for more advanced (systemic) treatment may reflect the fact that the parents had children who were attending secondary care and therefore were a subset of patients who either had not been managed adequately in primary care or required complex care. Not only should GP trainees learn how to use topical treatments such as emollients and topical corticosteroids, but they should also be aware of systemic treatments and, as both parents and healthcare professionals commented, know when to refer.

Parents in particular appreciated the role of the dermatology specialist nurse in the care of eczema patients and felt that the atopic eczema curriculum should include learning about the role of dermatology nurses. However the GP dermatology curriculum already recognises the vital role of nurses stating that “Our nursing colleagues too are a remarkable reservoir of knowledge, approaching patients with skin disease differently from GPs. Specialist health visitors or district nurses are also worth talking to, as of course is the specialist dermatology nurse practitioner.” Ideally GP trainees should learn from the expertise of dermatology nurses, just as they do from specialist nurses working in areas such as diabetes or asthma, and appreciate their role in eczema management.

Parents also highlighted the importance of addressing associated atopic conditions including food allergy so that appropriate advice is given. GP trainees should have sufficient knowledge to be able to explain atopic disease, to correct misconceptions and to avoid inappropriate restrictive diets (see parent’s comment in Box 3), but they should also be able to recognise when further investigation may be required.

In contrast to healthcare professionals, parents felt that GP trainees should know about alternative and complementary therapies for atopic eczema. The GP curriculum already recognises that many patients with chronic disorders pursue complementary and alternative medicine and the generic curriculum emphasises the importance of exploring patients’ beliefs and medication history.

### 4.2. How Should the Atopic Eczema Curriculum Be Implemented?

The RCGP curriculum document on “Care of people with Skin Problems” sets out a required knowledge base for GPs and mentions as a broad learning outcome the importance of being able to recognize and instigate appropriate treatment for common skin conditions such as eczema [10]. The equivalent curriculum statement on “Care of Children and Young People” does not specifically mention atopic eczema. The RCGP curriculum could be strengthened by including atopic eczema in both paediatric and dermatology sections as well as by providing internal links between these sections. Childhood atopic eczema should also feature in workplace-based assessments and MRCGP examinations.

Only a minority of GP trainees will undertake an approved hospital post in a dermatology department or have contact with dermatology nurses during their training. There are insufficient dermatologists, GPs with expertise in dermatology or dermatology nurses to train all GP trainees in the UK (more than 3000 enter the 3-year GP Speciality Training Programmes annually) [16,17], However, there are numerous opportunities for GP trainees to learn about and reflect on the challenges of managing atopic eczema under the supervision of their GP trainer, as this is an extremely common problem presenting to GPs and in many cases, could be managed appropriately in primary care.

The learning outcomes identified in this study should guide trainers as well as learners. Electronic learning modules, such as those provided by the BMJ, could be used to supplement learning in the workplace. Trainees could also draw on the guidance from NICE [5] as well as information provided by organisations such as the British Association of Dermatologists and the National Eczema Society.

### 4.3. Parents Are Potential Teachers

The parents of children with atopic eczema may also be a valuable teaching resource. Most patients appreciate an opportunity to contribute to doctors’ training and their teaching can generate new insights [18,19,20]. The parents of children with chronic atopic eczema, particularly those who have had access to secondary care and the support of dermatology nurses, have accumulated considerable first-hand “eczema expertise.” Their knowledge, stories and experience could provide the basis for memorable teaching. We recommend that GP trainers work in partnership with willing parents of children with chronic atopic eczema to deliver the curriculum and train the next generation of GPs.

## 5. Conclusions

This study identifies important learning outcomes on the management of atopic eczema that should be included in the RCGP curriculum. It also highlights the importance of patients and their parents as a valuable resource in the development of medical education.

## References

[1] Williams H.C. (1995). On the definition and epidemiology of atopic dermatitis. Dermatol. Clin..

[2] Fennessy M., Coupland S., Popay J., Naysmith K. (2000). The epidemiology and experience of atopic eczema during childhood: A discussion paper on the implications of current knowledge for health care, public health policy and research. J. Epidemiol. Commun. Health.

[3] Lewis-Jones S., Mugglestone M.A. (2007). Management of atopic eczema in children aged up to 12 years: Summary of NICE guidance. BMJ.

[4] Schofield J.K., Fleming D., Grindlay D., Williams H. (2011). Skin conditions are the commonest new reason people present to general practitioners in England and Wales. Br. J. Dermatol..

[5] National Institute for Health and Clinical Excellence Atopic eczema in children: Management of atopic eczema in children from birth up to the age of 12 years. www.nice.org.uk/CG057.

[6] Lewis-Jones S. (2006). Quality of life and childhood atopic dermatitis: The misery of living with childhood eczema. Int. J. Clin. Pract..

[7] Zuberbier T., Orlow S.J., Paller A.S., Taieb A., Allen R., Hernanz-Hermosa J.M., Ocampo-Candiani J., Cox M., Langeraar J., Simon J.C. (2006). Patient perspectives on the management of atopic dermatitis. J. Allergy Clin. Immunol..

[8] Ersser S.J., Latter S., Sibley A., Satherley P.A., Welbourne S. (2007). Psychological and educational interventions for atopic eczema in children. Cochrane Database Syst. Rev..

[9] Bieber T. (2008). Atopic dermatitis. N. Engl. J. Med..

[10] Royal College of General Practitioners (2010). Care of People with Skin Problems. http://www.rcgp.org.uk/gp-training-and-exams/~/media/Files/GP-training-and-exams/Curriculum-2012/RCGP-Curriculum-3-2013;21-Skin-Problems.ashx.

[11] General Medical Council Tomorrow’s Doctors. Outcomes and standards for undergraduate medical education. http://www.gmc-uk.org/Tomorrow_s_Doctors_1214.pdf_48905759.pdf.

[12] Dolan T.A., Lauer D.S. (2001). Delphi study to identify core competencies in geriatric dentistry. Spec. Care Dent..

[13] Clayton R., Perera R., Burge S. (2006). Defining the dermatological content of the undergraduate medical curriculum: A modified Delphi study. Br. J. Dermatol..

[14] Alahlafi A., Burge S. (2005). What should undergraduate medical students know about psoriasis? Involving patients in curriculum development: Modified Delphi technique. BMJ.

[15] Charman C.R., Morris A.D., Williams H.C. (2000). Topical corticosteroid phobia in patients with atopic eczema. Br. J. Dermatol..

[16] McHenry P.M., Williams H.C., Bingham E.A. (1995). Management of atopic eczema. Joint Workshop of the British Association of Dermatologists and the Research Unit of the Royal College of Physicians of London. BMJ.

[17] Centre for Workforce Intelligence (2011). Shape of the Medical Workforce: Informing Medical Specialty Training Numbers. http://www.cfwi.org.uk/publications/medical-shape-2011.

[18] National Recruitment Office for General Practice Training (2012). Annual Reports. Http://www.gprecruitment.org.uk/about.html.

[19] Howe A., Anderson J. (2003). Involving patients in medical education. BMJ.

[20] Wykurz G., Kelly D. (2002). Developing the role of patients as teachers: literature review. BMJ.

